# Perception Toward Cosmetic Surgeries Among Adults in Saudi Arabia and Its Associated Factors: A Cross-Sectional Study

**DOI:** 10.7759/cureus.64338

**Published:** 2024-07-11

**Authors:** Teef A Alqahtani, Ebtehal S Althagafi, Maha H Alsofiani, Raghad M Alasmari, Mohammed K Aljehani, Azza A Taha, Mohammad Eid M Mahfouz

**Affiliations:** 1 College of Medicine, Taif University, Taif, SAU; 2 College of Medicine, King Abdulaziz University, Jeddah, SAU; 3 Family and Community Medicine, Taif University, Taif, SAU; 4 Department of Surgery, College of Medicine, Taif University, Taif, SAU

**Keywords:** acceptance, perception, saudi arabia, body dysmorphic disorder, cosmetic surgery

## Abstract

Objective: This study assessed the acceptance of cosmetic surgery among the Saudi population and examined its relationship with body dysmorphic disorder and the participants' demographic characteristics.

Methods: A cross-sectional study was conducted between June 2023 and February 2024. A sample of 1368 participants were recruited from hospitals, clinics, and public places such as malls, parks, and universities and were asked to complete a self-administered, pre-validated, and open-access questionnaire about the presence of body dysmorphic disorder using the BDD scale and the acceptance of cosmetic surgery using the ACSS scale, analyzed using IBM Corp. Released 2015. IBM SPSS Statistics for Windows, Version 23.0. Armonk, NY: IBM Corp.

Results: More than half of the participants were female, single, and had a higher educational level. Only 3.0% of the participants had previously undergone cosmetic surgery (CS). Rhinoplasty was the most common cosmetic surgery performed by both genders. More than half of the participants indicated that they had concerns about their appearance, which caused them distress, torment, or pain. However, only one-third of the participants demonstrated acceptance toward CS. There was a significant positive correlation between body dysmorphic disorders (BDD) and acceptance of the cosmetic surgery scale (ACSS). Moreover, higher significant acceptance for CS (p-value is <0.001) was observed in females, south region, 32- to 40-year-olds, postgraduate degrees of education, married persons, and those undergoing previous cosmetic surgery.

Conclusion: Concerns about appearance causing distress were prevalent among participants, yet acceptance of cosmetic surgery was moderate. Individuals with higher levels of BDD symptoms are more inclined to view cosmetic surgery as a viable solution to their perceived concerns about their appearance. Moreover, being female, from the southern region, aged 32 to 40 years, with postgraduate education, married individuals, and having prior cosmetic surgery experience are motives to accept CS. These findings underscore the complex interplay between psychological factors and demographic characteristics in shaping the acceptance of cosmetic surgery.

## Introduction

Modern society has witnessed a recent upswing in cosmetic procedures due to a prevalent obsession with the achievement of youth and beauty, a greater influence on appearance, and the desire for self-improvement [1].

The American Society for Aesthetic Plastic Surgery estimates that between 2014 and 2015, spending on surgical and nonsurgical procedures rose by 1.5 billion dollars. In 2015, surgical interventions accounted for 58% of the overall cost, while nonsurgical procedures made up 42% [2]. 

Several factors contribute to the expansion of cosmetic surgery today, including medical advancements, patient characteristics, and media influences. For many years, the media has had a significant influence on personal appearance and decisions regarding cosmetic surgery [3].

Despite advancements in plastic surgery, the general population still lacks adequate understanding, particularly regarding cosmetic surgery. Furthermore, it is unknown how knowledgeable the medical profession is about cosmetic surgery. Public understanding of aesthetic plastic surgery will increase in proportion to the expertise of medical professionals who inform or misinform the public about these procedures, contributing to the public health system [4].

Cosmetic procedures, both surgical and nonsurgical, have experienced steady growth throughout the Americas, Europe, the Middle East, Asia, and Australia. The number of cosmetic surgeries (CS) in the United States has risen from about 15,000 in 1949 to 15.7 million in 2016. In 2018, cosmetic procedures accounted for 14.1% of all surgical procedures. However, CS faces negative stigmatization in some populations [5].

Unfavorable perceptions regarding cosmetic surgery exist in some societies. They criticize it as fake or artificial beauty. People in these societies give much concern to social recognition, and their behaviors are influenced by feeling respected as social individuals. They may consider that having CS will negatively impact their social reputation or social position in their societies. Despite these perceptions, people may choose cosmetic surgery due to factors that counterbalance negative acceptance of CS [6].

Modern society grapples with the cosmetic surgery paradox, influenced by sociopsychological and sociocultural attitudes toward appearance and body perceptions. These factors promote acceptance of cosmetic surgery despite risks and societal criticism. However, positive attitudes toward cosmetic surgery do not always lead to undergoing the procedure [7].

Numerous predictors for acceptance of cosmetic surgery in most populations have been identified by research and include body image, body dysmorphic disorder, body mass index, self-esteem, and psychological status. Moreover, demographic and cultural factors like gender, age, and race/ethnicity/culture may also have a role in CS acceptance [5]. 

Previously, cosmetic surgery was opposed in Saudi Arabia due to health risks and religious beliefs. Advancements in procedures and reassurance from family and friends have shifted attitudes toward its safety and acceptance [4].

In various societies, numerous studies have assessed the perceptions of the general public toward plastic and reconstructive surgery [8-10]. However, limited research has been carried out in the Middle East on this topic [11]. Consequently, the current study seeks to address this gap by evaluating the acceptance of cosmetic surgery in Saudi Arabia through a comprehensive survey and exploring the impact of body dysmorphic disorder and demographic characteristics on such acceptance.

## Materials and methods

Study design and setting: A descriptive cross-sectional study conducted from June 2023 to February 2024. Ethical approval No. (HAO-02-T-105) was obtained from Taif University to conduct the study. A comprehensive volunteer sampling approach was implemented across all regions of Saudi Arabia, covering the Northern, Southern, Western, Eastern, and Central regions. Within each region, participants were recruited voluntarily from various settings, including hospitals, clinics, and public places such as malls, parks, and universities. Saudi Arabia, the largest country in the Middle East and the fifth largest in Asia has a population of approximately 32 million, with 63% of the population under 30 years old [12]. The inclusion criteria included Saudi citizens who can read and write and are aged 18 years or older, with no specific exclusion criteria specified.

Sample size: The sample size of this study was calculated using the formula for cross-sectional studies [13]: n = z^2^p(1 − p)/d^2^, where n is the sample size, z = 1.96 for a confidence level (α) of 95%, p is the anticipated population proportion (expressed as a decimal) (50%) for the largest sample size, and d is precision (0.05 [5%]). The estimated sample size was 384. However, we received a high response from participants, resulting in 1368 complete questionnaires. We included all respondents in the analysis to enhance the study's reliability, statistical power, representativeness, and exploration of differences across various demographics. 

Tools of data collection: We used a pre-validated and self-administered Arabic questionnaire. The questionnaire consists of four main sections: (a) Consent, (b) demographic information, (c) the BDD (body dysmorphic disorder) questionnaire, which consists of seven questions about body dysmorphic disorder [14], and (d) the 15-item ACSS (Acceptance of Cosmetic Surgery Scale) [4], which measures interpersonal and social factors and considers acceptance and attitudes toward cosmetic surgery. The ACSS questionnaire includes five questions about each of the three subscales, which are intrapersonal, social, and consideration. All 15 questions are rated on a seven-point Likert scale, starting from 1 (strongly disagree) to 7 (strongly agree), except the number 10 question, which is reversely coded. Higher scores indicate more positive acceptance of CS.

Statistical analysis: The data from the questionnaire were entered into a database (Microsoft Excel for Mac, version 16.32), which requires cleaning before analysis using IBM Corp. Released 2015. IBM SPSS Statistics for Windows, Version 23.0. Armonk, NY: IBM Corp. Frequencies and percentages were calculated for the demographic characteristics of the study population, body dysmorphic disorders, and acceptance of cosmetic surgery. Normality of distribution was checked in SPSS using the Kolmogorov-Smirnov test, where ACSS showed a non-normal distribution among participants, so the relationship between demographic variables and ACSS was tested using the Mann-Whitney U test for the two categorical variables and the Kruskal-Wallis test for more than three categories of variables. The correlation between BDD and ACSS was tested using Spearman correlation; the p-value is considered significant if it is <0.05.

## Results

A total of 1368 participants completed the questionnaire. More than half of the participants were female, single, and had a higher educational level. The majority of participants were from the western region and were aged between 20 and 31 years (41.5% and 54.6%, respectively). More details about demographic information are shown in Table 1.

**Table 1:** Demographic information of the participants (N=1368)

Demographic information	Category	Frequency and Proportion n (%)
Gender	Male	446 (32.6%)
	Female	922 (67.3%)
Region	Western	569 (41.5%)
	Eastern	190 (13.8%)
	Central	436 (31.8%)
	North	44 (3.2%)
	South	129 (9.4%)
Age	Less than 20	229 (16.7%)
	20-31	748 (54.6%)
	32-40	196 (14.3%)
	41-50	137 (10.0%)
	More than 50	58 (4.2%)
Educational Level	Bachelor	896 (65.4%)
	High School	377 (27.5%)
	Post Graduate Degree	95 (6.9%)
Marital Status	Married	423 (30.9%)
	Single	945 (69.0%)

**Figure 1:**
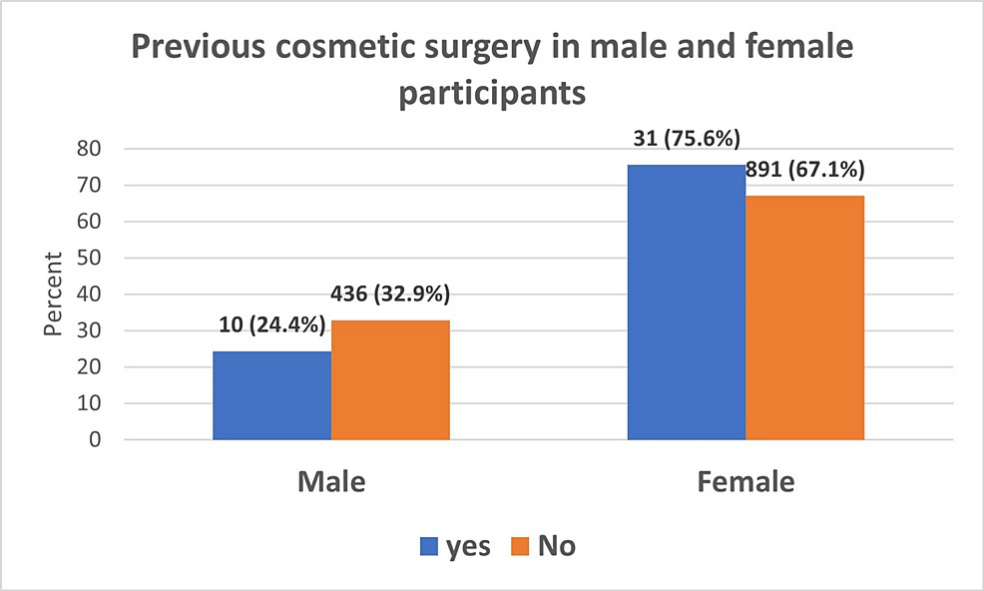
Prevalence of cosmetic surgery among male and female participants

**Figure 2:**
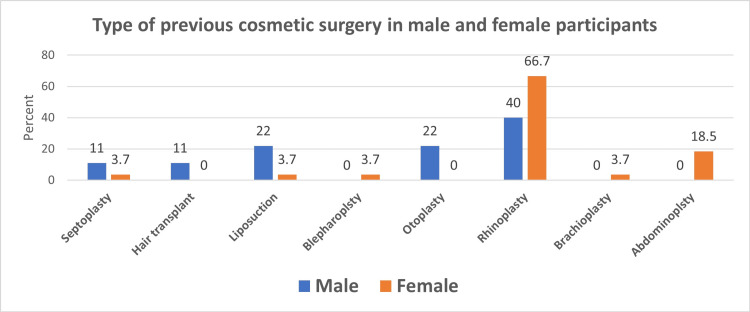
Distribution of the type of previous cosmetic surgery among male and female participants

Figures 1, 2 show that only 41 (3%) participants reported having previously undergone CS; the majority, 31 (75.6%) were female. The most common previous cosmetic surgery operation in both male and female participants was the rhinoplasty operation (4 (40%) in males and 18 (66.7%) in females. 

Tables 2, 3 present the detailed responses to the Body Dysmorphic Disorder scale questions among study participants. Approximately 34.6% (474 participants) expressed concerns about their appearance, with 27% (372 participants) reporting preoccupation and a desire to worry less about their appearance. Additionally, 34.6% (473 participants) indicated avoiding certain activities due to appearance-related concerns. More than half of the participants (54.3%, 743 individuals) reported appearance-related concerns causing distress, torment, or pain, while 36.2% (495 participants) noted impairment in social, occupational, or other important areas of functioning. Furthermore, 36.3% (496 respondents) reported significant interference in their social life due to appearance concerns, and 26% (356 participants) indicated interference with schoolwork, job performance, or other roles. Detailed responses are summarized in the below-mentioned tables.

**Table 2:** Body dysmorphic disorders (BDD) of the participants were presented in frequencies (n) and proportions (%) for the first three questions in the BDD questionnaire

Body Dysmorphic Disorders	Yes	No
Are you very worried about your appearance in any way?	474 (34.6%)	894 (65.4%)
Do these concerns preoccupy you? That is, you think about them a lot and wish that you could worry about them less?	372 (27.2%)	996 (72.8%)
Do you avoid doing anything because of your appearance concerns?	473 (34.6%)	895 (65.4%)-

**Table 3:** Body dysmorphic disorders (BDD) of the participants were presented in frequencies (n) and proportions (%) for the second four questions in the BDD questionnaire

Body Dysmorphic Disorders	No	Mild, not too disturbing	Moderate, disturbing but still manageable	Severe, very disturbing	Extreme, disabling
Do these concerns cause you a lot of distress, torment or pain? (select the best answer)	625 (45.7%)	418 (30.6%)	279 (20.4%)	28 (2%)	18 (1.3%)
Do these concerns cause you any impairment social, occupational or other important areas of functioning? (select the best answer)	873 (63.8%)	279 (20.4%)	171 (12.5%)	32 (2.3%)	13 (1%)
Do these concerns often significantly interfere with your social life? (select the best answer)	872 (63.7%)	269 (19.7%)	174 (12.7%)	33 (2.4%)	20 (1.5%)
Do these concerns often significantly interfere with your school work, job or ability to function in your role? (select the best answer)	1012 (74%)	195 (14.3%)	123 (9%)	24 (1.8%)	14 (1%)

Table 4 illustrates participants' acceptance of cosmetic surgery, summarizing overall agreement and disagreement percentages across 15 questionnaire items. Approximately 32.6% of participants expressed approval of cosmetic surgery, while 47.6% indicated disagreement with its acceptance.

**Table 4:** The acceptance of cosmetic surgery scale (ACSS) of the participants is presented in frequencies (n) and proportions (%) Q1: It makes sense to have a minor CS rather than spend years feeling bad about the way you look. Q2: CS is a good thing because it can help people feel better about themselves. Q3: In the future, I could end up having some kind of CS. Q4: People who are very unhappy with their physical appearance should consider CS as one option. Q5: If CS can make someone happier with the way they look, then they should try it. Q6: If I could have a surgical procedure done for free, I would consider trying CS. Q7: I have sometimes thought about having CS. Q8: If I knew there would be no negative side effects or pain, I would like to try CS. Q9: I would seriously consider having CS if my partner thought it was a good idea. Q10: I would never have any kind of plastic surgery. Q11: I would think about having CS in order to keep looking young. Q12: If it would benefit my career, I would think about having plastic surgery. Q13: I would seriously consider having cosmetic surgery if I thought my partner would find me more attractive. Q14: CS can be a big benefit to people's self-image. Q15: If a simple CS procedure would make me more attractive to others, I would think about trying it.

Items	Disagree a lot	Disagree somewhat	Disagree a little	Neutral	Agree a little	Agree somewhat	Agree a lot	Agreement %		
								Yes	No	
Q1	390 (28.5%)	172 (12.6%)	97 (7.1%)	308 (22.5%)	190 (13.9%)	116 (8.5%)	95 (6.9%)	29.3	48.2	
Q2	219 (16%)	100 (7.3%)	80 (5.8%)	336 (24.6%)	291 (21.3%)	186 (13.6%)	156 (11.4%)	46.3	29.1	
Q3	480 (35.1%)	109 (8%)	102 (7.5%)	249 (18.2%)	224 (16.4%)	122 (8.9%)	82 (6%)	31.3	50.6	
Q4	373 (27.3%)	160 (11.7%)	140 (10.2%)	332 (24.3%)	200 (14.6%)	106 (7.7%)	57 (4.2%)	26.5	49.2	
Q5	390 (28.5%)	128 (9.4%)	117 (8.6%)	287 (21%)	237 (17.3%)	113 (8.3%)	96 (7%)	32.6	46.5	
Q6	527 (38.5%)	124 (9.1%)	109 (8%)	278 (16.8%)	159 (11.6%)	108 (7.9%)	111 (8.1%)	27.6	55.6	
Q7	513 (37.5%)	115 (8.4%)	86 (6.3%)	208 (15.2%)	235 (17.2%)	117 (8.6%)	94 (6.9%)	32.7	52.2	
Q8	475 (34.7%)	124 (9.1%)	80 (5.8%)	201 (14.7%)	207 (15.1%)	130 (9.5%)	151 (11%)	35.6	49.6	
Q9	517 (37.8%)	123 (9%)	97 (7.1%)	273 (20%)	164 (12%)	85 (6.2%)	109 (8%)	26.2	53.9	
Q10	172 (12.6%)	120 (8.8%)	136 (9.9%)	369 (27%)	95 (6.9%)	87 (6.4%)	389 (28.4%)	41.7	31.3	
Q11	587 (42.9%)	113 (8.3%)	102 (7.5%)	238 (17.4%)	158 (11.5%)	97 (7.1%)	73 (5.3%)	23.9	58.7	
Q12	489 (35.7%)	113 (8.3%)	94 (6.9%)	259 (18.9%)	203 (14.8%)	100 (7.3%)	110 (8%)	30.1	50.9	
Q13	558 (40.8%)	115 (8.4%)	86 (6.3%)	242 (17.7%)	178 (13%)	96 (7%)	93 (6.8%)	26.8	55.5	
Q14	252 (18.4%)	67 (4.9%)	68 (5%)	274 (20%)	315 (23%)	204 (14.9%)	188 (13.7%)	51.6	28.3	
Q15	519 (37.9%)	135 (9.9%)	99 (7.2%)	234 (17.1%)	185 (13.5%)	95 (6.9%)	101 (7.4%)	27.8	55	
Total								32.7	47.64	

**Figure 3:**
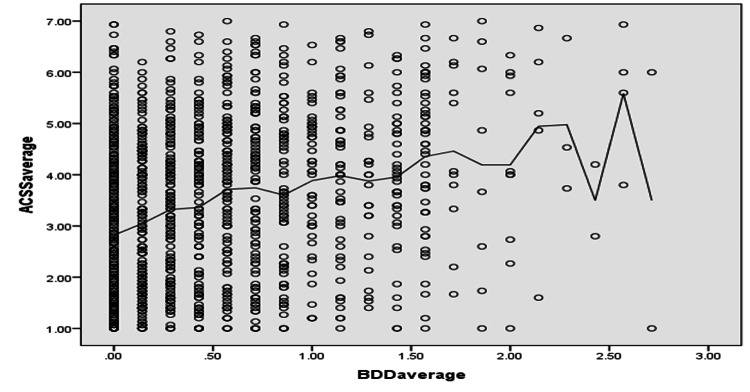
Correlation between body dysmorphic disorder (BDD) and acceptance of cosmetic surgery scale (ACSS)

The Spearman coefficient correlation analysis was conducted between BDD and ACSS in Figure 3, which showed a significant positive correlation between the Body Dysmorphic Disorder and Acceptance of Cosmetic Surgery Scale (r of Spearman's rho = 0.297 with a p-value < 0.001). 

The study identified significant associations across various demographic variables with acceptance of cosmetic surgery (CS). Higher acceptance rates were observed among females (p < 0.001), individuals residing in the south region (p = 0.012), those aged 32-40 years (p < 0.001), individuals with postgraduate education (p = 0.006), married individuals (p < 0.001), and those who had previously undergone cosmetic surgery (p < 0.001). Details are presented in Table 5.

**Table 5:** Association between demographic information and the acceptance of cosmetic surgery scale (ACSS) presented in Mean ± Standard Deviation The Mann-Whitney test was used for two categorical variables, whereas the Kruskal-Wallis test was used for more than three categories of variables.

Characteristics	Category	Mean ± SD	p-value
Gender	Male	3.1 ± 1.5	<0.001*
	Female	3.5 ± 1.6	
Regions	Western	3.3 ± 1.6	0.012*
	Eastern	3.4 ± 1.5	
	Central	3.2 ± 1.6	
	North	3.3 ± 1.6	
	South	3.8 ± 1.5	
Age	Less than 20	2.8 ± 1.6	<0.001*
	20-31	3.3 ± 1.5	
	32-40	3.9 ± 1.5	
	41-50	3.7 ± 1.6	
	More than 50	3.4 ± 1.8	
Education level	Bachelor	3.3 ± 1.5	0.006*
	High School	3.3 ± 1.6	
	Post Graduate Degree	3.8 ± 1.6	
Marital status	Married	3.6 ± 1.6	<0.001*
	Single	3.2 ± 1.6	
Previous cosmetic surgery	Yes	4.8 ± 1.3	<0.001*
	No	3.3 ± 1.6	

## Discussion

Several global studies have documented the recent progressive increase in the prevalence of cosmetic surgeries and subsequent increasing positive attitudes toward cosmetic procedures in different parts of the world. However, only limited studies from Arab countries have been found regarding this issue [15]. Our study assessed the prevalence of CS and investigated the attitudes and acceptance of the Saudi population toward cosmetic surgeries and whether body dysmorphic disorder and the other demographic characteristics of participants impact this acceptance. The sample of the current study consisted of a majority of participants aged between 20 and 31 years, with a predominance of females. Most of them were from the western region and had a bachelor's degree, with the majority of them being single.

According to the 2019 international survey on aesthetic and cosmetic procedures, among the top 30 countries reporting the highest rates of plastic surgery all over the world, Saudi Arabia ranks 29th [16], which indicates a low prevalence of CS in Saudi Arabia. This is obvious from our study, where only 3% of the participants reported having previously undergone CS. Similarly, a previous study at King Abdul Aziz University in Jeddah, Saudi Arabia, reported that only 2.2% of participants had undergone previous cosmetic surgery [17].

A gap exists between the positive attitude toward CS and the actual behavior of having it done. It is important to note that a positive attitude toward CS acceptance does not always translate into actually undergoing CS [7]. Son (2011) stated that only 26% of the participants who reported a positive attitude toward CS had undergone CS [18].

Alotaibi reported in 2021 that females constituted the vast majority of the population undergoing CS in all community populations studied [5]. In our study, most of the participants who underwent previous CS were females (75.6%), which aligns with the 2018 annual survey done by the International Society of Aesthetic Plastic Surgery, which reported that 86.5% of all the aesthetic CSs performed worldwide were done by women [19].

Rhinoplasty is one of the five most popular plastic surgeries, especially among females, which could be due to the significant effect of nose shape on facial beauty [20]. This agrees with our findings, where we found that the most preferred CS by both our male and female participants who underwent previous CS was a rhinoplasty operation (40% in males and 66.7% in females).

Body dysmorphic disorder (BDD) is a common underdiagnosed psychiatric disorder that is characterized by extreme preoccupation about a self-perceived defect, which could result in significant impairment of psychosocial functioning and translate to behaviors like avoiding social gatherings. Feelings of shame, depression, and even committing suicide are often associated with this condition [21]. We investigated the body dysmorphic disorder among our participants, and we found that more than half of the participants (54.3%) had concerns about their appearance, which caused them distress, torment, or pain, and more than a third of the participants were worried about their appearance. 34.6%, and the same percentage, reported avoiding certain things because of concerns about their appearance. Similarly, a previous study reported that 60.7% of the participants were very worried about their physical appearance, 32.5% felt preoccupied with these concerns, 22.9% reported a negative influence on their social life, 11.4% reported a negative impact on their schoolwork or job, and 50% declared they avoided doing things because of it [22].

The attitudes of participants toward cosmetic surgery are influenced by culture and religion. This can be seen in our study, where nearly half of the participants reported disagreement with accepting CS (47.6%), and the overall acceptance of cosmetic surgery in our study was 32.6%, indicating one-third approval of the CS among the participants. This result aligns with a previous study conducted in Saudi Arabia, which reported negative attitudes of the participants toward CS; only 33% of the study participants expressed approval toward CS [23]. These findings are not surprising, as religious issues and culture are considered major concerns and have a major influence on individual decisions in Saudi Arabia. This agrees with a previous UK study suggesting that “religiously conservative individuals of all faiths will have stricter views about “deception” and sins of vanity and will be less likely to undergo cosmetic surgery than more liberal or atheist individuals” [24]. However, a previous Saudi study by Al Doheyan and his colleagues was conducted in Al Riyadh and reported that the attitude of their study participants toward CS was reasonable. There was a general agreement among about 79% of the participants on the acceptance of cosmetic surgeries [17]. The high CS acceptance in that study could be justified by the special characteristics of the participants, who were female medical students.

Extensive research has documented the existence of an established relationship between acceptance of cosmetic surgery and psychological factors, including body image [25-27]. Similarly, our study showed that participants who experienced an increased BDD scale reported higher acceptance for CS, as was evident from the statistically significant positive correlation between Body Dysmorphic Disorder (BDD) and Acceptance of Cosmetic Surgery Scale (ACSS) (r of Spearman's rho = 0.297 with a p-value < 0.001).

Our study showed significant associations between acceptance for CS and female gender (p-value <0.001), south region (p-value 0.012), 32-40-year age category (p-value <0.001), postgraduate degree of education (p-value 0.006), married persons (p-value <0.001), and previous undergoing CS (p-value <0.001). Previous research mentioned that a greater willingness to consider cosmetic surgery is associated with many factors, including demographics like gender, ethnicity, and body image. The demographic factor that exerts the greatest impact on an individual's decision to have CS is gender. The available research consistently points out that females are more likely than males to approve of having cosmetic surgery [5,28]. In other areas around the world in which cosmetic surgeries are very popular, studies revealed that females had higher acceptance rates for cosmetic surgeries compared with males [29]. These results concur with our findings that females in our study had a significantly higher acceptance score for CS (3.5 ± 1.6) as compared to males (p-value <0.001). A possible explanation for this gender difference is the greater sociocultural pressures on women to achieve ideals of beauty and physical attractiveness.

South Korea is often referred to as the cosmetic surgery capital of the world. According to a study in South Korea, the majority of those undergoing cosmetic surgery are between 20 and 40 years old [30]. Our findings that the age category of 32 to 40 years has the highest acceptance score for CS align with that study. This high acceptance of CS in older subjects can be explained by the fact that people of increasing ages have a greater desire to slow their aging process, which influences their acceptance of CS.

Previous research declared that previous exposure to CS could result in a constant increase in their trial to improve self-appearance over time, known as CS addiction [7]. This is obvious in our study, where participants with a history of previously undergoing CS had a significantly better acceptance score for CS (p-value <0.001).

It is important to emphasize that our study provides valuable insights into the intersection of body dysmorphic disorder, acceptance of cosmetic surgery, and demographic factors, shedding light on factors influencing the acceptance of cosmetic procedures. However, the study's cross-sectional nature limits its ability to establish causal relationships between variables over time. Moreover, the limited number of male participants, especially those with post-graduate education backgrounds, potentially limits the generalizability of the findings.

## Conclusions

Concerns about appearance causing distress were prevalent among participants, yet acceptance of cosmetic surgery was moderate. Individuals with higher levels of BDD symptoms are more inclined to view cosmetic surgery as a viable solution to their perceived concerns about their appearance. Moreover, being female, from the southern region, aged 32 to 40 years, with postgraduate education, married individuals, and having prior cosmetic surgery experience are motives to accept CS. These findings underscore the complex interplay between psychological factors and demographic characteristics in shaping the acceptance of cosmetic surgery.

## References

[1] Honigman R, Castle DJ (2006). Aging and cosmetic enhancement. Clin Interv Aging.

[2] Dossett LA, Riesel JN, Griffin MR, Cotton BA (2011). Prevalence and implications of preinjury warfarin use: an analysis of the National Trauma Databank. Arch Surg.

[3] Brown A, Furnham A, Glanville L, Swami V (2007). Factors that affect the likelihood of undergoing cosmetic surgery. Aesthet Surg J.

[4] Morait SA, Abuhaimed MA, Alharbi MS, Almohsen BE, Alturki AT, Alarbash AA (2019). Attitudes and acceptance of the Saudi population toward cosmetic surgeries in Riyadh, Saudi Arabia. J Family Med Prim Care.

[5] Alotaibi AS (2021). Demographic and cultural differences in the acceptance and pursuit of cosmetic surgery: a systematic literature review. Plast Reconstr Surg Glob Open.

[6] Bonell S, Barlow FK, Griffiths S (2021). The cosmetic surgery paradox: Toward a contemporary understanding of cosmetic surgery popularisation and attitudes. Body Image.

[7] Kim Kim, S S (2022). What factors encourage the acceptance of cosmetic surgery? Differences in sociopsychological influences contingent upon cosmetic surgery experience. Fashion and Textiles.

[8] Sinno S, Barr J, Wilson S, Smith BD, Tanna N, Saadeh PB (2015). Public perceptions of plastic surgery: analysis and implications. J Craniofac Surg.

[9] Dunkin CS, Pleat JM, Jones SA (2003). Perception and reality—a study of public and professional perceptions of plastic surgery. Brit Jr Pla Surg.

[10] Conyard C., Schaefer N., Williams D. (2016). The understanding of plastic and reconstructive surgery amongst Queensland medical students. JPRAS Open.

[11] Almarghoub MA, Almarzouq SF, Alissa SI (2019). Public perception of plastic surgery in Saudi Arabia. Plast Reconstr Surg Glob Open.

[12] Saudi T (2019). Demographics of Saudi Arabia. Birth.

[13] Pourhoseingholi MA, Vahedi M, Rahimzadeh M (2013). Sample size calculation in medical studies. Gastroenterol Hepatol Bed Bench.

[14] Abdelhamid AS, Elzayat S, Amer MA, Elsherif HS, Lekakis G, Most SP (2023). Arabic translation, cultural adaptation, and validation of the BDDQ-AS for rhinoplasty patients. J Otolaryngol Head Neck Surg.

[15] AlShamlan NA, AlOmar RS, Al-Sahow AZ (2022). Cosmetic surgeries and procedures among youth in Saudi Arabia: a cross-sectional study of undergraduate university students in the Eastern Province. Postgrad Med J.

[16] (2024). ISAPS: international survey on aesthetic/cosmetic procedures. https://www.isaps.org/it/discover/about-isaps/global-statistics/reports-and-press-releases/global-survey-2019-full-report-and-press-releases-english/.

[17] Doheyan T.A., Al Saad A., Al Haidar A. (2016). Knowledge, attitude and practices concerning cosmetic surgery among female medical students at the University Hospital, King Saud University, Riyadh, Saudi Arabia. Brit Jr Med Med Res.

[18] Son E (2011). The differences of psychosocial characteristics according to plastic surgery experience and satisfaction with the plastic surgery. Korean J Woman Psychol.

[19] Takayanagi Takayanagi, S. S. (2024). ISAPS News: ISAPS’s survey and results-towards a new future. ISAPS.org.

[20] Zojaji R, Sobhani E, Keshavarzmanesh M, Dehghan P, Meshkat M (2019). The association between facial proportions and patient satisfaction after rhinoplasty: a prospective study. Plast Surg (Oakv).

[21] Murshidi R, Hammouri M, Al-Ani A (2024). Investigating the prevalence of body dysmorphic disorder among Jordanian adults with dermatologic and cosmetic concerns: a case-control study. Sci Rep.

[22] AlShahwan MA (2020). Prevalence and characteristics of body dysmorphic disorder in Arab dermatology patients. Saudi Med J.

[23] Bondagji MF, Sindi EE, Alamri GE (2024). Knowledge, attitudes, and practices with regard to cosmetic procedures among the general population in the Western Region of Saudi Arabia: a cross-sectional study. Cureus.

[24] Wenger NS, Carmel S (2004). Physicians' religiosity and end-of-life care attitudes and behaviors. Mou Sin Jr Med.

[25] Al Ghadeer HA, AlAlwan MA, AlAmer MA (2021). Impact of self-esteem and self-perceived body image on the acceptance of cosmetic surgery. Cureus.

[26] Swami V, Chamorro-Premuzic T, Bridges S, Furnham A (2009). Acceptance of cosmetic surgery: personality and individual difference predictors. Body Image.

[27] Di Gesto C, Nerini A, Policardo GR, Matera C (2022). Predictors of acceptance of cosmetic surgery: Instagram images-based activities, appearance comparison and body dissatisfaction among women. Aesthetic Plast Surg.

[28] Swami V, Hwang CS, Jung J (2012). Factor structure and correlates of the acceptance of cosmetic surgery scale among South Korean university students. Aesthet Surg J.

[29] Jovic M, Sforza M, Jovanovic M, Jovic M (2017). The Acceptance of Cosmetic Surgery Scale: Confirmatory factor analyses and validation among Serbian adults. Curr Psychol.

[30] Jin A, Whittall I (2022). A look at South Korean Plastic surgery. Exec Boa.

